# Baseline Study of Ultra-Clean Air Change Rate, Number, and Type of Microorganisms and Level of Particles During Trauma Surgery

**DOI:** 10.1177/19375867241302406

**Published:** 2024-12-05

**Authors:** J.L.A. Lans, N.M.C. Mathijssen, P.R. Goswami, J.J. van den Dobbelsteen, P.G. Luscuere, M. van der Elst

**Affiliations:** 1 Faculty Architecture and the Built Environment, 2860 Delft University of Technology, Delft, The Netherlands; 2 RHOC, 544197 Reinier Haga Orthopedic Center, Zoetermeer, The Netherlands; 3 Department of Orthopedic Surgery, 84744 Reinier de Graaf Hospital, Delft, The Netherlands; 4 Department of Medical Microbiology, 84744 Reinier de Graaf Hospital, Delft, The Netherlands; 5 541087 Faculty of Mechanical Engineering, 2860 Delft University of Technology, Delft, The Netherlands; 6 Department of Trauma Surgery, 84744 Reinier de Graaf Hospital, Delft, The Netherlands

**Keywords:** operating room, colony-forming units, infection prevention, ultra clean ventilation systems, instrument tables, surgical procedure

## Abstract

**Background:** The objective of an operating room (OR) ultra-clean ventilation system is to eliminate or reduce the quantity of dust particles and colony-forming units per cubic meter of air (CFU/m^3^). To achieve this, ultra-clean goal high air change rates per hour are required to reduce the particle load and number of CFU/m^3^. **Aim:** To determine the air quality in an ultra-clean OR during surgery, in terms of the number and type of microorganism and quantity of dust particles in order to establish a benchmark. **Methods:** Number of CFUs and the quantity of dust particles were measured. For measuring the CFUs, sterile extraction hoses were positioned at the incision, the furthest away positioned instrument table, and the periphery. At these locations, air was extracted to determine the quantity of dust particles. **Findings:** The number of CFU/m^3^ and particles was on average at wound level ≤1 CFU/m^3^ resp. 852.679 particles, at instrument table ≤1 CFU/m^3^ resp. 3.797 particles and in the periphery ≤8 CFU/m^3^, resp. 4.355 particles. **Conclusion:** The number of CFUs in the ultra-clean area is below the defined ultra-clean level of ≤10 CFU/m^3^ for ultra-clean surgery. The quantity of dust particles measured during surgery was higher than the defined ISO 5.

## Introduction

The objective of an operating room (OR) ultra-clean ventilation (UCV) system is to eliminate or reduce the quantity of dust particles and colony-forming units per cubic meter of air (CFU/m^3^). To achieve this goal, high air change rates per hour (ACH) are required to reduce the particle load and number of CFU/m^3^ (Langvatn et al., 2020; Lans et al., 2022).

The Dutch Federation of Medical Specialists (FMS) recently introduced a guideline for air handling in operating and treatment rooms (Kennisinstituut van de federatie van medisch specialisten, 2022). Only major orthopedic implant surgeries and major spinal surgeries (e.g., scoliosis) should be performed in a Class 1+ (Kennisinstituut van de federatie van medisch specialisten, 2022) OR. A Class 1+ OR corresponds to a Class 1 (DIN 1946-4: 2018-09, 2018; SIS-TS, 2015) OR according to international standards, where ultra-clean (HTM-03-01 Part A, n.d.; SIS-TS, 2015) air is defined as air which contains ≤10 CFU/m^3^. Other surgeries (Kennisinstituut van de federatie van medisch specialisten, 2022) could be performed in a generic OR with conventional (mixing) ventilation (CV) system with an air change rate of ≥20 h^−1^. This is in line with the WHO (World Health Organization, 2016) and for generic surgery with other international standards (DIN 1946-4: 2018-09, 2018; HTM-03-01 Part A, n.d.; NF S 90 351, 2013; Schweizerischer Verein von Gebäudetechnik-Ingenieuren, 2015; SIS-TS 39(E):2015, 2015). Evidence of the relation between higher ACH and a reduction of the number of surgical site infections (SSI) at most types of surgeries is weak (Kennisinstituut van de federatie van medisch specialisten, 2022; World Health Organization, 2016). Therefore, in accordance with their guideline, the FMS recommends a lower number of air changes per hour. Previous studies, during real (Agodi et al., 2015; Knudsen et al., 2021; Tammelin et al., 2023) or simulated surgery (Marsault et al., 2021), defined the air quality in terms of CFUs and sometimes dust particles directly and only underneath the Unidirectional Air Flow (UDAF) system and did not determine the measured number and type of microorganism close to the wound site and at the instrument table or in the periphery.

To date, most ORs in Dutch hospitals are built as an ultra-clean (Lans et al., 2023) OR. Since the recent FMS recommendation to reduce the number of air changes per hour for most surgeries, there is a need for a primary benchmark regarding the number and type of microorganisms and dust particles measured during real surgery at the wound site, instrument table, and periphery, measured in an ultra-clean OR. The present baseline study can be used as a contribution to the existing body of work (Knudsen et al., 2021; Marsault et al., 2021; Pedersen et al., 2019; Romano, Milani, Ricci, et al., 2020; Scaltriti et al., 2007; Tammelin et al., 2023) leading to a benchmark.

## Methods

**Figure 1.**
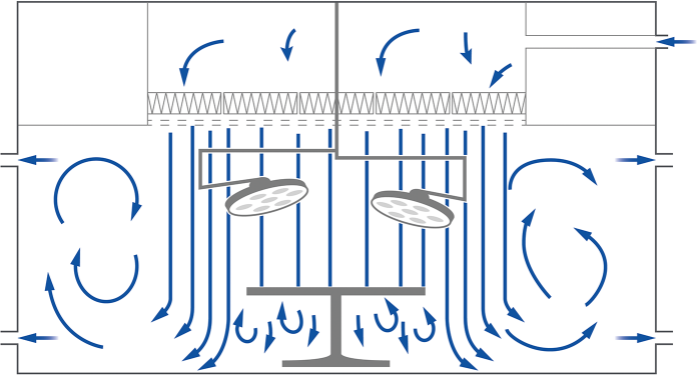
Working principle Unidirectional Air Flow (UDAF).

The measurements were executed in an OR at Reinier de Graaf Hospital (Delft, the Netherlands) during 29 different types of surgeries. The OR in this study was equipped with a UDAF system and classified as an ultra-clean OR class 1+. The UDAF system introduces the air directly (and only) above the protected area (see Figure 1). The staff present during surgery wore modern scrub suits made out of 99% polyester and 1% carbon fibers (Ljungqvist et al., 2015). The source strength using this type of clothing was 2.9 (0.9–5.7) CFU/s per person (Ljungqvist et al., 2015). The surface of the UDAF was 10.5 m^2^, and the total air volume introduced was 11.340 m^3^/h, which was 71 air changes per hour (ACH).

### Colony-Forming Unit Measurements

**Figure 2.**
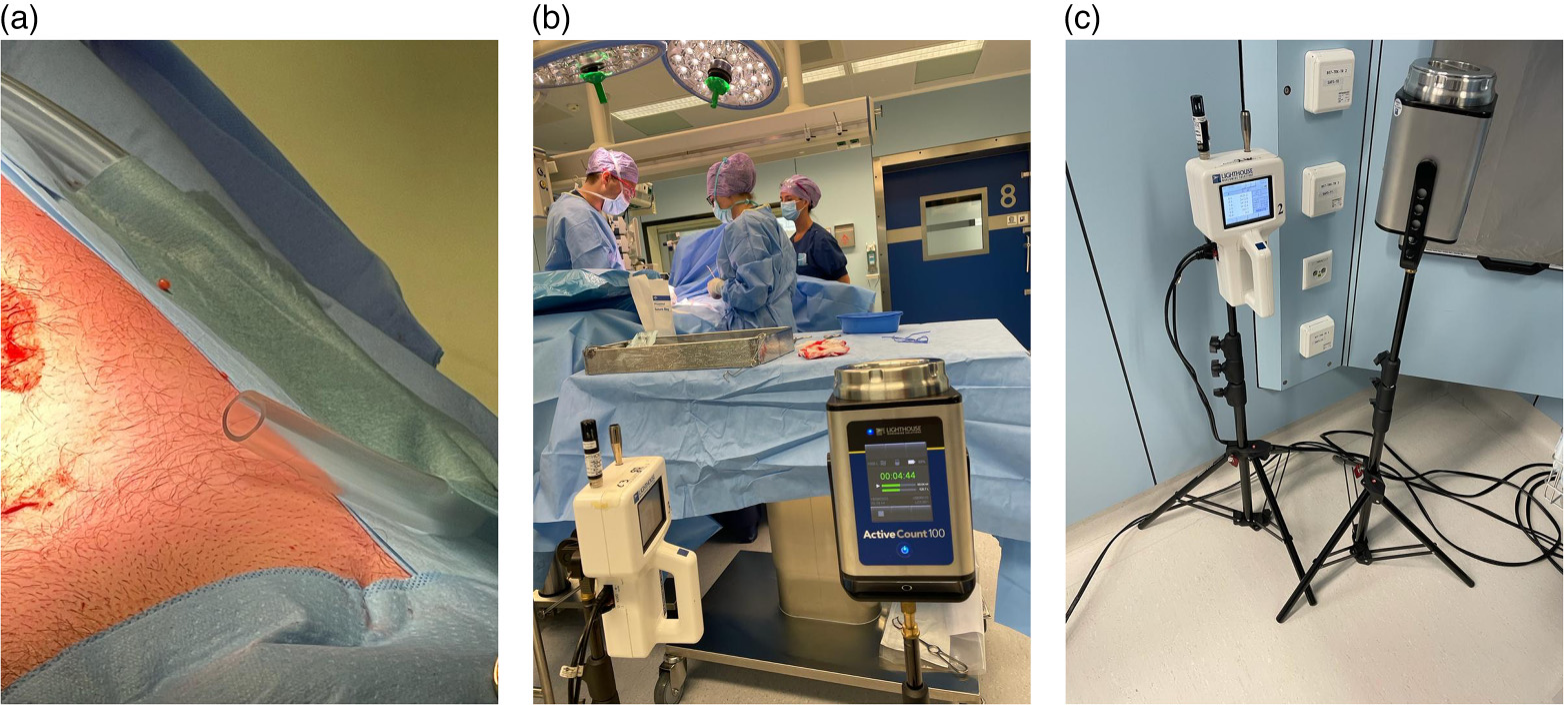
Photos of sample extraction hoses at the wound site (a), lighthouse 3016 handheld particle counters and portable lighthouse H100 active-air samplers during measurements at instrument table (b), and extraction point (c).

For the CFU measurements, sterile sample extraction hoses were positioned at three locations in the OR. The extraction hoses were positioned by the surgical staff at the location (<5 cm) of the incision, at the furthest away positioned instrument table from the surgical site and in the periphery close to an air extraction point (see Figure 2). The material of the sample tube was 1515 “python” neoprene hose dimensions Ø15 × 21(mm), temperature resistance: −20°C to +100°C. The maximum length of the sample hoses was 3 meters. Portable Lighthouse H100 active-air samplers were used for microbial air sampling, according to the slit principle. For 10 min, 100 dm^3^/min was sampled. Airborne bacteria-carrying particles were trapped via impaction on Tryptose Soy Agar cultivation plates. The plates were incubated aerobically for 2 × 24 h at 30–35°C. Directly after the incubation period, the hospital laboratory determined and reported the type of microorganism. The level of CFUs was assessed by means of the Swedish standard SIS—TS39:2015 (SIS-TS 39(E):2015, 2015). In contrary to other international standards (DIN 1946-4: 2018-09, 2018; Schweizerischer Verein von Gebäudetechnik-Ingenieuren, 2015), the focus in this standard (SIS-TS 39(E):2015, 2015) is on biological contamination and the use of microbiological methods for the assessment.

### Particle Count Measurements

The quantity of dust particles was measured at three positions in the OR. The quantity of dust particles was measured by the surgical staff at the location (<5 cm) of the incision, at the furthest away positioned instrument table from the surgical site and in the periphery close to an air extraction point (see Figure 2). For the particle measurements, Lighthouse 3016 handheld particle-counters with a flow rate of 2.83 l/min (0.1 ft^3^/min) were used. Particles with a size of ≥0.5 μm were measured. The ISO level of particles was assessed by means of the ISO 14644-1(ISO, 2015) standard, at-rest situation. At-rest is the condition where the OR or clean zone is complete with equipment installed and operating in a manner agreed upon, but with no personnel present (ISO, 2015).

During all measurements, the number of staff present, door openings, activity level, and extra ordinary occasions were recorded.

## Results

The number of CFU/m^3^ and particles during the surgeries was on average at wound level ≤1 CFU/m^3^ resp. 852,679 particles, at instrument table ≤1 CFU/m^3^ resp. 3,797 particles and in the periphery ≤8 CFU/m^3^, resp. 4,355 particles. The level of CFUs measured at the incision and instrument table was on average 0.5 resp. 0.7 CFU/m^3^. This is below the defined ≤10 CFU/m^3^ for ultra-clean surgery (SIS-TS 39(E):2015, 2015), see Table 1 for number of CFU/m^3^, type of microorganism, and quantity of dust particles measured.

**Table 1.** Descriptives operating room. Number (CFU/m^3^) and type of microorganism and quantity of dust particles measured. All surgeries were performed in the same OR equipped with an UDAF and in total 71 air changes per hour.

		Quantity particles, CFUs and measurement location									
		Wound			Instrument table			Periphery			
Type of surgery [duration]	Measurement cycle [10 min]	Mean quantity particles [0.5μm]	Mean level of CFU [CFU/m^3^]	Type of microorganism	Mean quantity particles [0.5μm]	Mean level of CFU [CFU/m^3^]	Type of microorganism	Mean level of CFU [CFU/m^3^]	Mean level of CFU [CFU/m^3^]	Type of microorganism	Remarks
Distal humeral fracture [105 min]	1	74,193	0	Not determined	2954	0	Not determined	1477	3	Not determined	
	2	23,532	1	Not determined	1092	0	Not determined	3853	3	Not determined	
	3	1413	0	Not determined	193	0	Not determined	1894	2	Not determined	
	4	32	0	Not determined	32	0	Not determined	514	4	Not determined	
	5	835	0	Not determined	1798	0	Not determined	449	4	Not determined	
	6	12,521	0	Not determined	1027	0	Not determined	0	20	Not determined	
	7	1702	2	Not determined	3403	0	Not determined	0	4	Not determined	
	8	385	0	Not determined	1926	1	Not determined	0	9	Not determined	
	9	88	0	Not determined	417	0	Not determined	0	5	Not determined	
	10	32	0	Not determined	4848	0	Not determined	0	11	Not determined	
	11	424	1	Not determined	3602	0	Not determined	0	13	Not determined	
	12	247	0	Not determined	2825	0	Not determined	1024	6	Not determined	
	13	4077	0	Not determined	424	0	Not determined	417	3	Not determined	
	14	64	0	Not determined	0	0	Not determined	1926	12	Not determined	
Laparoscopic inguinal hernia repair [30 min]	1	7544	0	Not determined	1284	0	Not determined	3114	8	Not determined	
	2	2440	0	Not determined	803	2	Not determined	3853	8	Not determined	
Open inguinal hernia repair [30 min]	3	69,634	0	Not determined	6967	0	Not determined	2215	17	Not determined	
	4	117,341	0	Not determined	0	3	Not determined	161	3	Not determined	
	5	67,419	0	Not determined	289	0	Not determined	1124	4	Not determined	
Elbow fracture [30 min]	6	81,160	0	Not determined	2761	0	Not determined	4398	4	Not determined	
	7	59,907	0	Not determined	64	2	Not determined	3692	6	Not determined	
Open inguinal hernia repair [30 min]	1	1,157,230	0	Not determined	264	0	Not determined	5072	15	Not determined	
	2	13,933	0	Not determined	0	0	Not determined	1637	26	Not determined	
Laparoscopic inguinal hernia repair [30 min]	3	9471	0	Not determined	0	0	Not determined	1284	3	Not determined	
	4	54,545	0	Not determined	0	0	Not determined	1284	8	Not determined	
Laparoscopic inguinal hernia repair [30 min]	5	4495	0	Not determined	0	0	Not determined	1637	2	Not determined	
	6	4559	0	Not determined	0	0	Not determined	1220	0	Not determined	
Open inguinal hernia repair [45 min]	1	10,113	0	Not determined	32	2	Not determined	1188	3	Not determined	
	2	8540	0	Not determined	32	4	Not determined	1156	1	Not determined	
	3	5169	0	Not determined	10,017	3	Not determined	15,603	4	Not determined	
Open inguinal hernia repair [30 min]	4	1,414,930	0	Not determined	3660	0	Not determined	2472	9	Not determined	
	5	161	0	Not determined	10,402		Not determined	5554	5	Not determined	
Plate osteosynthesis clavicle fracture [30 min]	6	14,961	2	Not determined	0	2	Not determined	771	1	Not determined	C-arm used during surgical procedure
	7	2321	0	Not determined	3114	0	Not determined	11,301	2	Not determined	
Laparoscopic inguinal hernia repair [30 min]	1	35,989	0	None	3371	0	None	1059	3	1× *Staphylococcus caprae* 1× *Micrococcus luteus* 1× *Staphylococcus epidermidis*	
	2	11,012	0	None	3307	1	1× *Staphylococcus epidermidis*	674	23	13× *Staphylococcus epidermidis* 8× *Micrococcus luteus* 2× *Bacillus* sp	
Patellar fracture repair [45 min]	3	325,216	0	None	74,771	0	None	15,121	17	6× *Micrococcus luteus* 6× *Staphylococcus epidermidis* 1× *Bacillus* sp 4× *Staphylococcus warneri*	C-arm used during surgical procedure
	4	1926	0	None	3307	0	None	0	3	1× *Staphylococcus capitis* 1× *Micrococcus luteus* 1× *Staphylococcus epidermidis*	
	5	8957	0	None	6325	0	None	963	10	5× *Micrococcus luteus* 3× *Staphylococcus haemolyticus* 2× *Staphylococcus epidermidis*	
	6	289,773	0	None	5532	0	None	1830	10	5× *Micrococcus luteus* 5× *Staphylococcus haemolyticus*	
Ankle fracture fixation [45 min]	7	3,177,296	0	None	4225	2	2× *Micrococcus luteus*	5121	41	1× *Bacillus* sp 16× *Micrococcus luteus* 20× *Staphylococcus epidermidis* 4× *Staphylococcus capitis*	C-arm used during surgical procedure
	8	78,976	0	None	2538	3	2× *Corynebacterium* sp 1× *Micrococcus luteus*	21,285	6	4× *Micrococcus luteus* 2× *Staphylococcus warneri*	
Open inguinal hernia repair [90 min]	1	2,613,831	2	1× *Micrococcus luteus* 1× *Staphylococcus hominis*	128	0	None	83,118	3	Not determined	
	2	867	0	None	64	0	None	3018	6	Not determined	
Laparoscopic inguinal hernia repair [90 min]	3	12,360	0	None	32	0	None	3178	10	Not determined	
	4	35,315	2	1× *Micrococcus luteus* 1× *Staphylococcus epidermidis*	7159	1	1× *Staphylococcus warneri*	9663	8	Not determined	
Laparoscopic inguinal hernia repair [45 min]	5	1,148,947	0	None	5040	0	None	5458	2	Not determined	
	6	61,833	0	None	0	0	None	2921	3	Not determined	
	7	55,637	0	None	0	0	None	4697	4	Not determined	
Removal of implants of the ankle [10 min]	1	5,648,228	0	None	22,345	0	None	15,057	7	Not determined	
Ankle fracture fixation [50 min]	2	4,109,118	0	None	5426	0		1316	0	Not determined	
	3	2,278,310	0		6421	8	1× *Staphylococcus epidermidis* 7× *Micrococcus luteus*	2279	0	Not determined	
	4	7,045,822	5	3× *Staphylococcus aureus* 2× *Staphylococcus pettenkoferi*	18,010	10	1× *Paracoccus yeei* 3× *Staphylococcus hominis* 5× *Kocuria rhizophila* 1× *Acinetobacter lwoffii*	8861	2	Not determined	
Laparoscopic inguinal hernia repair [45 min]	5	25,809,339	0		835	0	None	10,370	2	Not determined	
	6	2,689,437	1	1× *Paenibacillus urinalis*	96	0	None	13,869	5	Not determined	
	7	33,388	0		64	0	None	9278	1	Not determined	
Removal of lipoma swelling [10 min]	1	318,185	0	None	193	0		11,879	21	1× *Staphylococcus epidermidis* 1× *Moraxella osloensis* 1× *Micrococcus luteus*	
Removal of lipoma swelling [10 min]	2	9760	1	1× *Moraxella osloensis*	11,012	0	None	5394	19	1× *Staphylococcus vitulinus* 1× *Staphylococcus hominis* 1× *Micrococcus luteus* 1× *Moraxella osloensis*	
Laparoscopic inguinal hernia repair [50 min]	3	8,857,047	0	None	5137	0	None	0	14	1× *Staphylococcus epidermidis* 1× *Paracoccus yeei* 1× *Moraxella osloensis* 1× *Corynebacterium simulans* 1× *Micrococcus luteus*	
	4	110,567	0	None	12,103	0	None	0	17	1× *Staphylococcus hominis* 1× *Acinetobacter johnsonii* 1× *Staphylococcus warneri*	
	5	14,415		None	3981	0	None	0	19	1× *Staphylococcus epidermidis* 1× *Staphylococcus aureus* 1× *Staphylococcus hominis* 1× *Micrococcus luteus*	
	6	1605	0	None	4495	0	None	0	12	1× *Staphylococcus hominis* 1× *Moraxella osloensis* 1× *Mirococcus luteus*	
Laparoscopic inguinal hernia repair [35 min]	7	68,831	0	None	4141,4	0	None	417,4	7	1× *Staphylococcus epidermidis* 1× *Micrococcus luteus* 1× *Moraxella osloensis* 1× *Kocuria rhizophila*	
	8	33,581	1	1× *Micrococcus luteus*	4173,6	0	None	32,1	8	1× *Staphylococcus hominis* 1× *Staphylococcus petrasii* 1× *Micrococcus luteus* 1× *Corynebacterium amycolatum*	
	9	5522	1	1× *Staphylococcus hominis*	2889	0	None	0	9	1× *Staphylococcus epidermidis* 1× *Micrococcus luteus* 1× *Moraxella osloensis*	
Ankle fracture fixation [10 min]	1	31,526	0	None	24,496	0	None	18,877,3	1	1× *Staphylococcus epidermidis*	
Laparoscopic inguinal hernia repair [60 min]	2	321,813	0	None	257	0	None	995,2	1	1× *Micrococcus luteus*	
	3	7994	0	None	0	0	None	160,5	1	1× *Staphylococcus epidermidis*	
	4	13,323	0	None	0	0	None	545,8	5	1× *Moraxella osloensis* 2× *Micrococcus luteus* 1× *Staphylococcus saprophyticus* 1× *P. scleromae*	
	5	125,913	0	None	0	0	None	642,1	1	1× *Staphylococcus warneri*	
	6	36,631	0	None	482	0	None	385,3	3	2× *Corynebacterium propinquum* 1× *Micrococcus luteus*	
	7	33,196	1	1× *Micrococcus luteus*	0	0	None	1380,5	0	None	
	8	33,099	0		963	0		866,8	0	None	
Laparoscopic inguinal hernia repair [30 min]	9	16,598	0	None	0	0	None	577,9	0	None	
Clavicle plate removal/fracture [20 min]	1	0	3	1× *Bacillus simplex* 1× *Micrococcus luteus* 31× grams of positive rods not determinable	0	1	1× *Micrococcus luteus*	64,2	8	3× *Staphylococcus epidermidis* 3× *Staphylococcus hominis* 1× *Micrococcus luteus* 1× grams of positive rods not determinable	
	2	0	2	1× *Cellulosimicrobium cellulans* 1× *Paenibacillus* sp	0	1	1× *Staphylococcus capitis*	0	3	2× *Staphylococcus hominis* 1× *Micrococcus luteus*	
Fracture fixation of ankle fracture [30 min]	3	56,680	10	4× *Corynebacterium minutissimum* 3× *Staphylococcus epidermidis* 1× *Escherichia coli* 1× *Enterococcus casseliflavus* 1× *Bacillus pumilus*	0	2	2× *Micrococcus luteus*	2439,9	20	3× *Staphylococcus epidermidis* 1× *Staphylococcus warneri* 3× *Micrococcus luteus* 9× *Staphylococcus warneri*	
	4	51,701	0	None	0	0	None	0	20	1× *Staphylococcus haemolyticus* 6× *Staphylococcus saprophiticus* 8× *Micrococcus luteus*	
	5	11,159	3	1× *Escherichia coli* 2× *Staphylococcus capitis*	0	0	None	0	46	4× *Paracoccus yeei* 26× *Staphylococcus warneri* 4× *Micrococcus luteus*	
Laparoscopic inguinal hernia repair [20 min]	6	29,276	1	3× *Micrococcus luteus*	0	0	None	n.a.	6	3× *Staphylococcus epidermidis* 3× *Micrococcus luteus*	
	7	181,588	0	None	0	4	2× *Staphylococcus hominis* 2× *Micrococcus luteus*	n.a.	4	2× *Micrococcus luteus* 1× *Staphylococcus epidermidis* 1× *Staphylococcus capitis*	
Mean		852,679	0,5		3797	0,7		4355	7,9		

## Discussion

Reducing energy consumption in hospitals has a high priority (Friedericy et al., 2019; Zarzycka et al., 2019). One possibility to save energy is to reduce the ACH in the OR (Lans et al., 2024; Loomans et al., 2020). For new construction projects, the choice is sometimes made to lower OR classifications (Kennisinstituut van de federatie van medisch specialisten, 2022), to lower the ACH, and to modify the existing air handling installation fitted in existing ORs (Lans et al., 2024). The WHO (World Health Organization, 2016) and FMS (Kennisinstituut van de federatie van medisch specialisten, 2022) stated that existing research on OR ventilation systems is flawed and that there is only weak evidence that OR ventilation systems help to reduce the incidence of SSIs (Brandt et al., 2008; Breier et al., 2011; Weinstein & Bonten, 2017). Other studies declare the opposite (Langvatn et al., 2020; Surial et al., 2022; Whyte & Lytsy, 2019) and advise to use a UCV system for infection-prone surgeries (HTM-03-01 Part A, n.d.; NICE guideline, 2020; SIS-TS 39(E):2015, 2015), where artificial implants are used.

This baseline study has been performed to enable a comparison regarding the number of CFU/m^3^, type of microorganism, and quantity of dust particles in an ultra-clean (HTM-03-01 Part A, n.d.; SIS-TS 39(E):2015, 2015) OR. Since the recent FMS advice to reduce the ACH for most surgeries, it is important to have a benchmark regarding the number and type of microorganisms and particles measured during surgery at the wound site, instrument table, and periphery, measured in an OR Class 1+.

In this study, there was a broad variety of microorganisms cultured at different locations, often there was little correlation between types of organisms found during one operation at different locations or in subsequent uses of the OR. In general, it can be said that an overwhelming majority of the cultured bacteria are not known as primary pathogens, such as *Staphylococcus aureus* (Bode et al., 2010). The majority of cultured organisms in this study are known as colonizing bacteria of the human skin (*Staphylococcus hominis, S. epidermidis, S. capitis*) which could pose a risk for low-grade prosthetic infections but were found in this study distant from the operating table to form a risk to the patient. Determination of the type of microorganisms shows a paucity of primary pathogens, with the largest numbers of cultured bacteria members of human colonizers or environmental contaminants that occasionally participate in prosthetic infections, and in this study in an OR equipped with a UDAF, were found distant from the site of the surgery to form a threat in those special cases. Besides the known SSI prevention measures (Seidelman et al., 2023), an ever-increasing air change rate is a concept that is on most occasions already beyond a reasonable expectation dose/response effect as long as the six general strategies supported by randomized trials are followed for prevention of SSIs: avoiding razors for hair removal, decolonization with intranasal antistaphylococcal agents and antistaphylococcal skin antiseptics for high-risk procedures, use of chlorhexidine gluconate and alcohol-based skin preparation, maintaining normothermia to keep the body temperature warmer than 36 °C, perioperative glycemic control, and use of negative pressure wound therapy.

During the measurements, the UCV system did not meet the at-rest ISO class 5 (DIN 1946-4: 2018-09, 2018; Kennisinstituut van de federatie van medisch specialisten, 2022) standards at the measuring locations during surgery. This is consistent with previous studies (Marsault et al., 2021; Scaltriti et al., 2007). Our study showed that the desired ISO5 (DIN 1946-4: 2018-09, 2018; Kennisinstituut van de federatie van medisch specialisten, 2022; NF S 90 351, 2013) classification was exceeded on every measurement location despite the high ACH (Langvatn et al., 2020). The quantity of particles measured during surgery was on average ISO8 at the wound site and ISO6 at the instrument table and in the periphery (ISO, 2015). This is consistent with other studies (Landrin et al., 2005; Stålfelt et al., 2023).

When changing the air change rate in an existing OR or when building a new OR, the selection of the classification of the OR and ACH should depend on the type of the surgical procedure. As well the number of people present, the heat load in the OR, the clothing procedure (Ljungqvist et al., 2015) etc. are important criteria. The impact of reducing the ACH on the measured numbers of CFUs and particles cannot be determined without considering the aforementioned parameters.

This study has several limitations.

First, the impact on SSIs when reducing the air change rate (Lans et al., 2024) in the OR is not determined. To date, most ORs in Dutch hospitals are designed as ultra-clean ORs (FMS Class 1+). Changing from an UCV to a CV air supply system can have an effect on the use of surgical smoke and contaminant removal effectiveness (Romano, Milani, Gustén, et al., 2020). However, reducing the air change rate will decrease the energy consumption of the OR air handling installation (Gormley et al., 2017; Marsault et al., 2021). A study is recommended to evaluate the impact on the number of SSIs and cultured bacteria when reducing the ACH according to the FMS. This study is recommended despite the fact that the outcomes will be influenced by parameters such as number and the behavior of staff present (Humphreys, 2018; Wang et al., 2020), number of door openings (Perez et al., 2018; Roth et al., 2019; Smith et al., 2013), amount of air introduced (Lans et al., 2022), type of surgical clothing (Cao et al., 2021; Ljungqvist et al., 2015), and type of surgical procedures (Friedericy et al., 2024).

Secondly, the number of staff present, door openings, activity level, and extraordinary occasions has influence on the number of CFU during surgery. Although we recorded these, we did not assess the impact of those activities on we did not assess the impact of those activities or occasions on the number of CFUs or the quantity of dust particles (Perez et al., 2018; Roth et al., 2019; Smith et al., 2013; Teter et al., 2017).

Third, in the current study, we only examined one hospital OR location. It is recommended to conduct a similar study in other hospitals and ORs where room geometry (Memarzadeh & Jiang, 2004), the ACH (Langvatn et al., 2020; Lans et al., 2024), and type of UCV system vary (Lans et al., 2022). The merging of all these data will give a more comprehensive picture of whether it is needed to apply high ACH.

In conclusion, the level of CFUs in the ultra-clean surgical area is below the standards defined ultra-clean level for ultra-clean ORs. The quantity of dust particles measured during surgery was higher than the standards defined ISO5 (ISO, 2015) at-rest. Regarding the number of particles (≥0.5μm) during surgery ISO8 (ISO, 2015) levels were reached at the wound site and ISO6 (ISO, 2015) at the instrument table and in the periphery.

## Implications for Practice

- It is relevant to understand how air quality in the OR affects the patient's risk of developing a SSI and the risk to staff when exposed to surgical smoke and other harmful substances.
- The design of the OR air handling installation and selection of the type of UCV system is important. The type of surgery should be considered when selecting the type of air handling and OR air supply system.
- When an OR is equipped with an ultra-clean air supply system that is protecting only the surgical site it will leave, the periphery of the OR at risk for contamination.
- All environmental parameters should be assessed to determine if a higher or lower ACH will benefit the asepsis of the OR.
